# Identification of avian polyomavirus and its pathogenicity to SPF chickens

**DOI:** 10.3389/fmicb.2023.1320264

**Published:** 2024-01-03

**Authors:** Tianshu Zhai, Jiajia Yan, Jia Wang, Dongni Kong, Lidan Hou, Yong Deng, Guoqian Gu, Tuanjie Wang, Xi Wang, Qinghong Xue, Chunsheng Yin, Jia Cheng, Guanlong Xu, Yaqing Mao

**Affiliations:** ^1^China Institute of Veterinary Drug Control, Beijing, China; ^2^College of Veterinary Medicine, Shanxi Agricultural University, Taigu, China; ^3^Department of Infectious Diseases and Public Health, Jockey Club College of Veterinary Medicine and Life Sciences, City University of Hong Kong, Kowloon, Hong Kong SAR, China; ^4^College of Animal Science and Technology, Jiangxi Agricultural University, Nanchang, China

**Keywords:** avian polyomavirus, biological identification, SPF chicken, pathogenicity, VP4

## Abstract

The research aimed to study an Avian polyomavirus strain that was isolated in Shandong, China. To study the pathogenicity of APV in SPF chickens, and provide references for epidemiological research and disease prevention and control of APV. The genetic characterization of APV strain (termed APV-20) was analyzed and the pathogenicity of APV was investigated from two aspects: different age SPF chickens, and different infection doses. The results revealed that the APV-20 exhibits a nucleotide homology of 99% with the other three APV strains, and the evolution of APV In China was slow. In addition, the APV-20 infection in chickens caused depression, drowsiness, clustering, and fluffy feathers, but no deaths occurred in the infected chickens. The main manifestations of necropsy, and Hematoxylin and Eosin staining (HE) showed that one-day-old SPF chickens were the most susceptible, and there was a positive correlation between viral load and infection dose in the same tissue. This study showed that SPF chickens were susceptible to APV, and an experimental animal model was established. This study can provide a reference for the pathogenic mechanism of immune prevention and control of APV.

## Introduction

Avian polyomavirus (APV) disease, also known as budgerigar fledgling disease (BFD). The disease is an important disease of parrots in birds (parrots, parakeets) caused by Aves polyomavirus type 1, and is a member of the polyomavirus family (Bernier et al., 1981), has been proven to be an acute infectious disease caused by APV. Genomic analysis of strains isolated from different birds revealed a high degree of sequence similarity (Stoll et al., 1993; Phalen et al., 1999), which led to the idea that the name APV should be used for all known isolates rather than the misleading BFD virus (Johne and Müller, 1998). APV can infect a wide range of birds, including parrots and geese. Especially to the young with a very strong pathogenicity, can cause a severe inflammatory response, resulting in the death of infected chicks (Ogawa et al., 2006).

Avian polyomavirus (APVs) have a 40–50 nm diameter non-enveloped icosahedral capsid and encode a double-stranded circular DNA genome of approximately 5,000 bp in length (Wang et al., 2022). The early region encodes the large Tumor antigen and the small Tumor antigen. The late region encodes the structural proteins VP1, VP2, and VP3, as well as two newly identified proteins, VP4 and VP4-delta (formerly known as Agnoprotein 1a and Agnoprotein 1b) (Wang et al., 2022), which are present only in APVs but not in mammalian polyomaviruses (Johne and Müller, 2001). The absence of VP4 also reduces the virulence and infectivity of the virus, indicating that VP4 may be an important factor leading to acute disease (Johne et al., 2007). VP4-delta is also found only in APV, and both VP4 and VP4-delta play an important role in APV replication (Liu and Hobom, 2000).

Avian polyomavirus (APV) has a wide range of hosts, and young parrots are the most important natural hosts for APV and have the most severe disease symptoms. The virus can also infect a variety of other avian species, such as geese and pigeons (Li et al., 2019). Compared with mammalian polyomavirus, APV shows unique biological characteristics, especially its ability to cause acute lesions in different organs of infected birds. The susceptibility and mortality of avian polyomycosis vary widely and are related to age, environmental health, stressors, or secondary diseases. Adult birds are tolerant and generally only have subclinical infection, with asymptomatic infections predominating, and although these birds have no clinical signs, the virus can be detected in their excreta and blood (Phalen et al., 1993, 2000).

At present, there are no effective control measures for avian polyomavirus. Most studies on the disease have focused on parrots, but APV has been isolated from infected chicken in the previous study, researchers had initially established a chicken model, 4 days of age chickens were used for virus infection test and the tissue culture infective dose was a power of ten lower in the earlier study (Fitzgerald et al., 1996). This indicates that chickens are an animal species that is vulnerable to APV. Hence, it is imperative to undertake more comprehensive research of the pathogen. Thus, to close the knowledge gap on the virus, infection models involving chickens are employed. In this study, an APV was isolated from infected parrots. To better understand the molecular characteristics of the identified strain, sequencing analysis was performed and a phylogenetic tree was constructed based on its complete genome. The pathogenicity of APV from two perspectives: varying infection doses and SPF chickens of varying ages. It serves as the foundation for the pathophysiology, early diagnosis, and prevention of this disease.

## Materials and methods

### Virus isolation and identification

In 2020, dozens of budgerigars died at a commercial farm in Shandong, China. The budgerigars showed rapid weight loss and exhibited heart enlargement, liver enlargement with hemorrhages. Heart, liver, and Kidney were collected from dead budgerigars. To identify the APV virus, the samples were ground and centrifuged. DNA/RNA was extracted (AxyPrep Body Fluid Viral DNA/RNA Miniprep Kit, Axygen, Corning, China) from the supernatants and then identified using primers. According to the APV genome sequence published in GenBank, the primers and probes used for TaqMan MGB probe fluorescence real-time quantitative PCR were designed using the Primer5 software (listed in Table 1). A TaqMan MGB probe-based, fluorescence real-time quantitative PCR was established to identify the APV virus. This isolated strain was designated APV-20.

**TABLE 1:** TaqMan probe real-time fluorescence quantitative PCR primers.

	Primers (5′-3′)
qVP4	Forward: ACCCTGCGCCAGGAATTAAA
	Reverse: GGGTGGTAGCAGTAGGGGTA
probe	5′-FAM-CATCTGCAGGCAGACGTAGATGATC-MGB-3′

### Virus sequencing and phylogenetic analysis

To sequence the full genome of the isolated APV, six pairs of primers covering the full genome of the APV were designed using the Primer5 software (listed in Table 2). The amplified PCR products were sequenced by the HUADA Company (Beijing, China). A dataset containing the sequences of 23 representative APV reference strains and used to construct the phylogenetic trees. Sequences were aligned using MAFFT v7.450 (Katoh et al., 2002). Maximum likelihood (ML) phylogenetic trees were inferred with IQ-Tree (v2.0.3) (Nguyen et al., 2015) using the best-fit nucleotide substitution model (TrNef + I + G4) and visualized using Fig Tree (v1.4.4). Phylogenetic support was estimated using 1,000 ultrafast bootstrap replicates.

**TABLE 2:** The whole genome of APV-specific amplification primers.

	Primers (5′-3′)
APV-1	Forward: CGGATATGCCCATGTTTGT
	Reverse: GAAGTTGCTGCTGTTTGCT
APV-2	Forward: CGACTAACGCAACCGAACC
	Reverse: GACAGCCTCCCACATAAGC
APV-3	Forward: CGTAAAGTCAGGTCCAGATAG
	Reverse: GCCCTTCCATACCCTCATA
APV-4	Forward: CCTACCACGCTATTTCAGT
	Reverse: GACCATGACAAGGGATGA
APV-5	Forward: GGTCTTACCCGTATTCACTG
	Reverse: TTGCCACTTGCTTATTACCT
APV-6	Forward: TTTTCGTCCACAACATTCA
	Reverse: GTAGGCGTTCCGTTAGGC

### Establishment of APV-infected CEF cell model and determination of virus titer

To study the pathogenicity of the APV20 in cells and chickens, chick embryo fibroblasts (CEF) cells were prepared and maintained in medium 199 supplemented with 10% fetal bovine serum. A total of 100 μl of virus solution was inoculated on the well-growing monolayer CEF cells and incubated in an incubator at 37°C for 2 h for virus absorption, and then the supernatant was replaced with medium 199 supplemented with 2% fetal bovine serum. The model was successfully established when the cytopathic effect (CPE) of 80% of cells was observed daily. Then, the virus titer (median tissue culture infective dose, TCID_50_) of APV was quantified using the Spearman–Kärber method (Spearman, 1908; Kärber, 1931; Lei et al., 2021).

### Determination of replication dynamics in CEF cells

Chick embryo fibroblasts (CEF) cells were spread on 24-well cell culture plates with a 2.0 × 10^5^ cells/healthy density to get the virus growth curve. When the cells grew to about 80%, APV was applied to infect CEF cells at 0.1MOI, and a Mock control group was set up. The cells and supernatant were harvested at 24, 48, 72, 96, and 120 h, respectively. After the viral DNA was extracted, the contents of VP4 and β-actin were detected by real-time quantitative PCR to determine the replication of APV and establish the growth curve of APV-infected CEF (The primer sequence is shown in Table 3). To further confirm the identification of virus infection, immunofluorescence assay (IFA) was detected using APV-VP4 specific monoclonal antibody.

**TABLE 3:** Real-time fluorescent quantitative PCR primers.

	Primers (5′-3′)
VP4	Forward: ACCCTGCGCCAGGAATTAAA
	Reverse: GGGTGGTAGCAGTAGGGGTA
β-actin	Forward: CCTAGCACAATGAAAATCAAGATCA
	Reverse: TCATCACAGGGGTGTGGGT

### Animal trials for pathogenicity analyses of the APV

To assess the pathogenicity of APV, we purchased specific pathogen free (SPF) chickens from Beijing Boehringer Ingelheim Viton Biotechnology Co., LTD. The experiment was divided into two parts. To study the infection degree of APV-20 in chickens of different days of age and the infection situation of chickens under different challenge doses. In Part I, 40 SPF chickens were randomly divided into four test groups (10 chickens per group; each group, respectively, 1, 5, 10, 20 day age of chickens); 20 SPF chickens were randomly divided into four control groups (5 chickens per group; each group, respectively, 1, 5, 10, 20 day age of chickens). Each chicken in test groups was intramuscularly

injected with 0.1 mL APV-20 virus containing 10^6^.^0^TCID_50_/ml. The control groups were mock-inoculated chickens, each chicken in groups was intramuscularly injected with 0.1 mL normal saline. In Part II, 40 SPF chickens were randomly divided into four test groups (10 chickens per group; each group was 1-day-old age of chickens); 20 SPF chickens were randomly divided into four control groups (5 chickens per group; each group was 1-day-old age of chickens). Each chicken in test groups was intramuscularly injected with 0.1 mL APV-20 virus, the virus content was 10^6^.^0^TCID_50_/ml, 10^5^.^0^TCID_50_/ml, 10^4^.^0^TCID_50_/ml, 10^3^.^0^TCID_50_/ml, respectively. The control groups were mock-inoculated chickens, each chicken in groups was intramuscularly injected with 0.1 mL normal saline reared.

## Results

### Genetic analysis of APV-20 full genome

**FIGURE 1:**
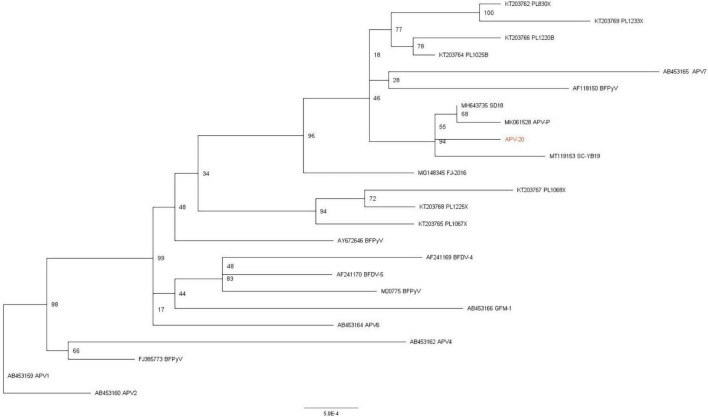
Phylogenetic and recombination analysis of APV. Phylogenetic tree based on APV whole genome with 23 reference APV strains. Phylogenetic support was estimated using 1,000 ultrafast bootstrap replicates.

In phylogenetic trees of full genome, compared with the consensus sequence of all APV reference strains. It showed that there are 23 representative APV reference strains to be used to construct the phylogenetic trees, and the similarity of the genome sequence between APV-20 and he domestic SD18, SC-YB19 and APV-P strains was more than 99%, and the evolutionary distances in the phylogenetic tree were very short (0.005), which suggests that the evolution of APV in China was slowly. The genotypes of APV in China formed a single branch and were closely related to Polish, Japanese, and American isolates (Figure 1).

### Virus biological identification

**FIGURE 2:**
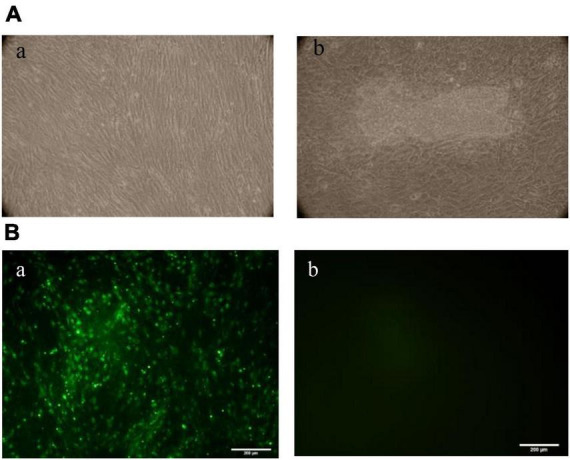
**(A)** Observation of CEF lesions after APV infection. The cell aggregation, accumulation, wrinkling, and rupture without wall attachment cytopathic changes were observed in the infected group on day 7. The unvaccinated cell group showed no cytopathic changes. (a) Control group, (b) APV-infected group. **(B)** Immunofluorescence assay (IFA) results of CEF cells infected with APV. IFAs showing the reactivity of a monoclonal antibody against APV VP4 protein to APV-20 strain infected at 7 days. (a) CEF cells infected with APV-20; (b) Uninfected CEF cells (20×).

Based on the virus’s biological features, the cytopathic changes became more noticeable on day 7. These changes mostly showed up as cells clumping together, wrinkling, breaking apart without attaching to the wall, and other damage. In contrast, the unvaccinated cell group showed no cytopathic changes. The cells grew tightly and in good condition (Figure 2A). The results showed that the cell infection model was successfully established on CEF, and APV could replicate effectively on CEF. The viral titer of APV was 10^6.875^TCID_50_/0.1 mL, measured by the Spearman–Kärber method. The IFA results showed that the cytoplasm of infected CEF cells showed green positive fluorescence staining, while there was no fluorescence signal in uninfected CEF cells (Figure 2B).

### Determination of viral replicate kinetics in CEF cells

**FIGURE 3:**
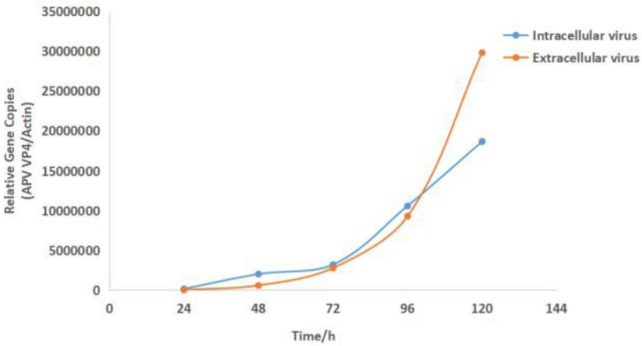
Growth curve results of the virus APV-20. Growth kinetics of APV-20 strain on CEF cells.

Compared with the virus-uninfected group, the relative quantification of APV in infected cells and supernatant at different time points was analyzed, and the one-step growth curve of the intracellular and extracellular virus was drawn. At 24 h after infection, the virus particles could be detected inside and outside the cells. At 24–72 h after infection, the intracellular and extracellular virus content increased slowly, and the trend was the same. After 72 h, the content of intracellular and extra-cellular viruses increased logarithmically, but the growth rate of extracellular viruses was faster than that of intracellular viruses. At 120 h, the intracellular virus content was lower than the extracellular (Figure 3).

### Pathogenicity in SPF chickens

There was no mortality in either the infected or the control groups during the entire experimental procedure. No clinical signs were observed in the control group. In 14 days, the infected chickens showed loss of appetite, loose feathers, spirit depression, and lethargy. The chickens were euthanized at the end of the trial, and samples of the heart, liver, lungs, kidneys, and bursa of Fabricius were collected to assess the viral load in these organs and observe organ lesions. The lesioned tissues were H&E stained. The statistical results are shown in Tables 4, 5.

**TABLE 4:** Observation of organ lesions.

Group		Observation of organ lesions				Infections
		Bursa	Heart	Kidney	Liver	
Different days of age	1-day-old	Atrophy and hemorrhage	Cardiomegaly	Hemorrhage	enlarged, hemorrhage and a red and yellow marble-like pattern	+
	5-day-old	Hemorrhage	Cardiomegaly	/	/	+
	10-day-old	/	/	/	Enlarged	+
	20-day-old	/	/	/	Enlarged	+
Different dose APV infection	10^6.0^TCID_50_	Atrophy and hemorrhage	Cardiomegaly	Severe congestion	Swollen and yellowed with blood stasis	+
	10^5.0^TCID_50_	/	/	Hemorrhage, swollen	Swollen with marginal stasis	+
	10^4.0^TCID_50_	/	/	Hemorrhage, swollen	/	+
	10^3.0^TCID_50_	/	/	Hemorrhage, swollen	/	+

This table counts and compares the observation of tissue and organ lesions of chickens infected with APV virus at different ages and with different doses of APV virus.

**TABLE 5:** Histopathological observation.

Group		Histopathological observation				Infections
		Bursa	Heart	Kidney	Liver	
Different days of age	1-day-old	germinal centers were lost, hyperplasia of connective tissue	Infiltration of macrophages and lymphocytes, interstitial myocardial hemorrhage	Interstitial nephritis, protein exudation from the lumen of the renal tubules, glomerulus atrophy	intranuclear inclusions, granulomatous inflammation	+
	5-day-old	germinal centers were lost, hyperplasia of connective tissue	Infiltration of macrophages and lymphocytes	Interstitial nephritis, protein exudation from the lumen of the renal tubules	intranuclear inclusions, granulomatous inflammation	+
	10-day-old	germinal centers were lost, hyperplasia of connective tissue	Infiltration of macrophages and lymphocytes	Interstitial nephritis	Granulomatous inflammation	+
	20-day-old	germinal centers were lost	Infiltration of macrophages and lymphocytes	Interstitial nephritis	Granulomatous inflammation	+
Different dose APV infection	10^6.0^TCID_50_	germinal centers were lost, hyperplasia of connective tissue	Infiltration of macrophages and lymphocytes, interstitial myocardial hemorrhage	Interstitial nephritis, protein exudation from the lumen of the renal tubules, glomerulus atrophy	Granulomatous inflammation, a large number of lymphocytic infiltrates	+
	10^5.0^TCID_50_	germinal centers were lost, hyperplasia of connective tissue	Infiltration of macrophages and lymphocytes	Interstitial nephritis	Swollen with marginal stasis	+
	10^4.0^TCID_50_	germinal centers were lost	Infiltration of macrophages and lymphocytes	Interstitial nephritis	Granulomatous inflammation	+
	10^3.0^TCID_50_	germinal centers were lost	/	Interstitial nephritis	/	+

This table counts and compares the histopathological observation of chickens infected with APV virus at different ages and with different doses of APV virus.

**FIGURE 4:**
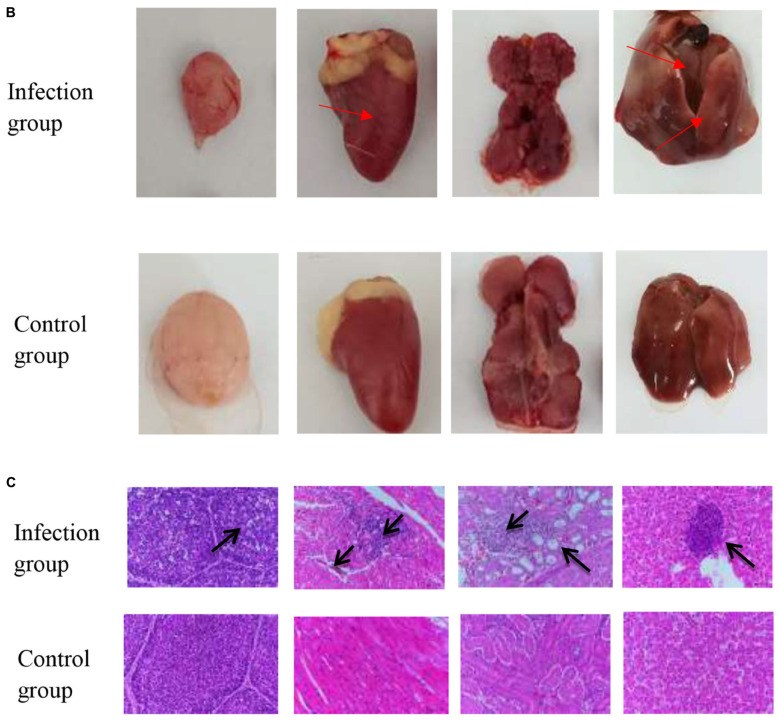
**(A)** The viral load of different tissues of SPF chickens infected with APV at different ages. The APV can infect multiple organs, and virus loads in 1-day-old chickens are significantly higher than in other ages. (APV)APV-20 infected group; (MOCK) uninfected control group. Significant differences were considered when *P < 0.05, ***P < 0.001, *⁣*⁣**P < 0.0001. **(B)** Gross observation of organs from 1-day-old SPF chickens infected with APV. Necropsy lesions observation of Bursa, Heart, Kidney, and Liver on the 1-day-old. SPF chickens infected with APV. The pathological features were observed in multiple organs. **(C)** Histological sections of 1-day-old SPF chickens infected with APV showing typical lesions. Histopathological Observation of Bursa, Heart, Kidney, and Liver on the 1-day-old SPF chickens infected with APV. The pathological features were observed in the sections of multiple organs.

**FIGURE 5:**
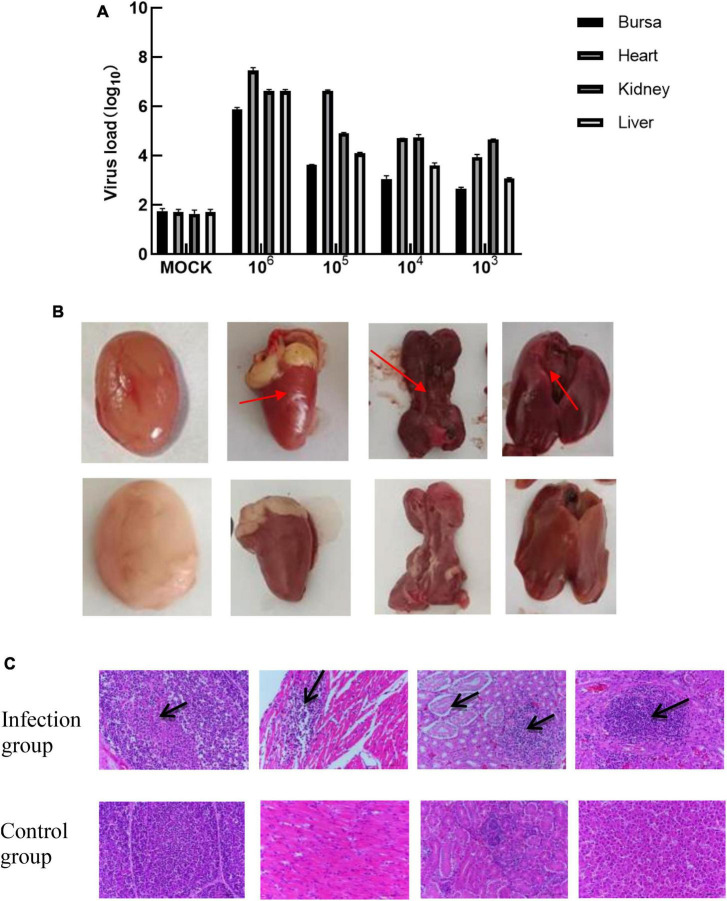
**(A)** The viral load of different tissues of SPF chickens infected with different doses of APV. The pathological features were observed in the sections of multiple organs. (Infection group) Bursa, heart, kidney, and liver of the 1-day-old infected group. (Control group) Bursa, heart, kidney, and liver of the 1-day-old uninfected group. **(B)** Observation of necropsy lesions after SPF chickens infected with 10^6.0^TCID_50_/0.1 mL doses of APV. (Infection group) Bursa, heart, kidney, and liver of the 1-day-old infected group. (Control group) Bursa, heart, kidney, and liver of the 1-day-old uninfected group. **(C)** Histopathological Observation of Bursa, heart, kidney, and live in SPF chickens infected with 10^6.0^TCID_50_/0.1 mL t doses of APV. (Infection group) Bursa, heart, kidney, and liver of 10^6.0^TCID_50_/0.1 mL infected group. (Control group) Bursa, heart, kidney, and liver of the uninfected group.

The tissues of the control group and the infected group at 1, 5, 10, and 20 days of age and 4 infective dose groups were detected by Taq-Man PCR. Test results showed that viruses with high copy numbers could be detected in the bursae of Fabricius, heart, kidney, and liver tissues, and 1-day-old chickens were the most susceptible to APV (Figure 4A). There was a positive correlation between viral load and infected dose in the same tissues of the four infected groups; the higher the infected dose, the higher the viral load in the tissues. The highest viral load in different tissues was in the heart and kidney, followed by the liver, and the bursa of Fabricius was lower. No virus was detected in the control group (Figure 5A).

Macroscopic observation of the lesions of the tissues revealed the presence of normal lung tissue in the control group and significant lesions in the bursa of Fabricius, the kidney, and the liver of chickens in the APV-20-inoculated group. Chickens in the infected group mainly showed apparent atrophy and hemorrhage in the bursa, severe hemorrhage in the kidney, enlarged and yellowish liver with petechial hemorrhage, and a red and yellow marble-like pattern (Figures 4B, 5B). H&E staining indicated that the germinal centers of bursal lymphoid nodules were lost, hyperplasia of connective tissue, interstitial myocardial bleeding, cell loss, lymphocyte infiltration, glomerular atrophy Interstitial nephritis of varying degrees was observed. No noticeable pathological changes were observed in the control group (Figures 4C, 5C). In this experiment, the pathogenicity of APV in chickens was studied from two aspects of infecting SPF chickens of different ages and different infective doses. The results showed that the susceptibility of chickens to APV varied with age. Among them, 1-day-old SPF chickens were the most susceptible, and the older the experimental animals were, the less sensitive they were. The reason may be that the immune system develops gradually and becomes more resistant to viruses. Among the four different infection doses, the higher the infection dose, the more severe the clinical symptoms, necropsy changes, and histopathological observations of SPF chickens. The challenge dose was set as 10 ^6^.^0^TCID_50_/0.1 mL per chicken to ensure stable results of the challenge test. It was confirmed that APV was significantly pathogenic to SPF chickens, the susceptible age of APV to SPF chickens was determined, and the challenge dose of APV was used as the challenge virus.

## Discussion

Viruses in the genus Polyoma belong to DNA viruses and have a large number of hosts, including a variety of vertebrates, such as birds, rodents, monkeys, and humans (Us, 2013). In contrast to mammalian polyomavirus, APV exhibits distinctive biological attributes, most notably its capacity to induce acute lesions across various organs in avian hosts. According to the results of phylogenetic trees analysis, APV evolved slowly in China. Although APV is widespread, not many gene sequence in GenBank, and most of the sequences were usually from Japan and Poland, as well as two genome sequences from China. In the phylogenetic tree of the full genomes, the APV-20 isolated in this study was with high identity to strains from Poland, Japan and USA.

Avian polyomavirus (APV) is especially highly pathogenic to chicks and can cause acute infection in multiple organs of the body, mainly with an inflammatory response (Davis et al., 1981). Histopathological observations reveal inclusion bodies in several organs, amphophilic to basophilic inclusion bodies with a clearing of the centre. Feather follicles may show hyperkeratosis (Herder et al., 2011). There is monocyte and inflammatory cell infiltration in the interstitial spaces of cardiac myocytes, with coagulative necrosis in the visual field’s foci, and some calcium deposits (Gough, 1989). The liver had areas of necrosis, with monocytes and heterophils infiltrating around the confluent area infiltration and vacuolar degeneration of some hepatocytes (Jacobson et al., 1984). Mononuclear cell infiltration is common in the vessel wall and around the vessels, again containing inclusions, being present in the walls of some of the larger blood vessels (Randall et al., 1987). Pulmonary lesions are less common and are usually stasis (Rossi et al., 2005).

Some studies reported that ballooning change and septicemia were also observed, which were judged to be the result of bacterial infection at the same time (Roy et al., 2004). Xia et al. (2000) used the isolated APV-purified virus to conduct animal experiments on 20-day-old psittacus chicks. Psittacus died throughout the experiment in the subcutaneous, intramuscular, and oral injection groups, yet the times of onset and death were prolonged. Liver and lung congestion, splenomegaly, swollen kidneys, and liver hemorrhage are the most frequent lesions found in deceased chicks. The global chicken industry is developing quickly, and it produces chicken on a huge scale in groups. For animal regression experiments, chicken is a convenient animal because it is vulnerable to APV.

In this study, no mortalities were recorded in all experimental trials with different ages, and doses of infection. Through the establishment of animal models of infected chickens, it was found that the mortality of APV in chickens was not high. But even at very low doses, viral infections can still be detected in the bursae of Fabricius, heart, liver, and kidney. This indicates that chickens are highly susceptible to APV as recipient animals. The clinical symptoms, necropsy changes, histopathological changes, and tissue viral load of infected SPF chickens were analyzed one-day-old SPF chickens were the most susceptible, and the older the experimental animals were, the less susceptible they were. The absence of mortality despite the high dose experimental infection and the heart affection. The reason may be that the immune system developed gradually and the resistance to the virus increased. In four different dose groups, chickens in the low-dose group also showed symptoms of viral infection. These results indicate that chickens are highly susceptible as receptor animals. We speculate that the experiment was conducted in a negative pressure isolation device, and the feeding environment, such as density, ventilation conditions, and sanitary conditions, will affect the severity of the disease, and the secondary infection of bacteria, such as *Escherichia coli* or Pasteurellosis, will also aggravate the disease (Sugiyama et al., 2010). However, whether APV can induce co-infection with other viruses and lead to severe clinical symptoms remains to be studied.

## Conclusion

In conclusion, an APV strain APV-20 was isolated in Shandong, China. The full genome of APV-20 was determined and analyzed. And we completed the study of the pathogenicity of APV. SPF chickens showed symptoms of depression, clumps, and fluffy and messy feathers after infection. A total of 14 days after infection, the autopsy showed different degrees of macroscopic lesions in the bursa of Fabris, heart, kidney, and liver. Pathological lesions of the bursae of Fabricius, heart, kidney, and liver were detected, and viral infection was detected. We demonstrated the significant pathogenicity of APV to SPF chickens, determined the susceptible age of APV to SPF chickens, as well as the challenge dose of APV. Our results provided the experimental basis for the diagnosis, epidemiological research, and control of APV.

## Data availability statement

The datasets presented in this study can be found in online repositories: NCBI GenBank, BankIt2776605 APV-20 PP057981.

## Ethics statement

The animal study was approved by the Animal Welfare Committee of China Institute of Veterinary Drug Control. The study was conducted in accordance with the local legislation and institutional requirements.

## Author contributions

TZ: Conceptualization, Data curation, Formal analysis, Investigation, Methodology, Software, Writing – original draft, Writing – review and editing. JY: Conceptualization, Data curation, Investigation, Methodology, Software, Writing – original draft, Writing – review and editing. JW: Software, Writing – review and editing. DK: Formal analysis, Writing – review and editing. LH: Formal analysis, Writing – review and editing. YD: Formal analysis, Writing – review and editing. GG: Software, Writing – review and editing. TW: Formal analysis, Writing – review and editing. XW: Formal analysis, Writing – review and editing. QX: Formal analysis, Writing – review and editing. CY: Formal analysis, Writing – review and editing. JC: Formal analysis, Writing – review and editing. GX: Data curation, Formal analysis, Writing – review and editing. YM: Conceptualization, Formal analysis, Funding acquisition, Writing – review and editing.
