# Correction: Identification of avian polyomavirus and its pathogenicity to SPF chickens

**DOI:** 10.3389/fmicb.2026.1847058

**Published:** 2026-05-13

**Authors:** Tianshu Zhai, Jiajia Yan, Jia Wang, Dongni Kong, Lidan Hou, Yong Deng, Guoqian Gu, Tuanjie Wang, Xi Wang, Qinghong Xue, Chunsheng Yin, Jia Cheng, Guanlong Xu, Yaqing Mao

**Affiliations:** 1 China Institute of Veterinary Drug Control, Beijing, China; 2 College of Veterinary Medicine, Shanxi Agricultural University, Taigu, China; 3 Department of Infectious Diseases and Public Health, Jockey Club College of Veterinary Medicine and Life Sciences, City University of Hong Kong, Kowloon, Hong Kong SAR, China; 4 College of Animal Science and Technology, Jiangxi Agricultural University, Nanchang, China

**Keywords:** avian polyomavirus, biological identification, SPF chicken, pathogenicity, VP4

In the published article, there was an error in [Figure 4B] as published. [In Figure 4B, the macroscopic distribution of organs does not correspond to the histological images in Figure 4C (specifically liver and kidney).]. The corrected [Gross observation of organs from 1-day-old SPF chickens infected with APV.] and its caption ^**^[Observation on necropsy lesions of the 1-day-old SPF chickens infected with APV. The pathological features were observed in multiple organs.] appear below.

**Figure 4B:**
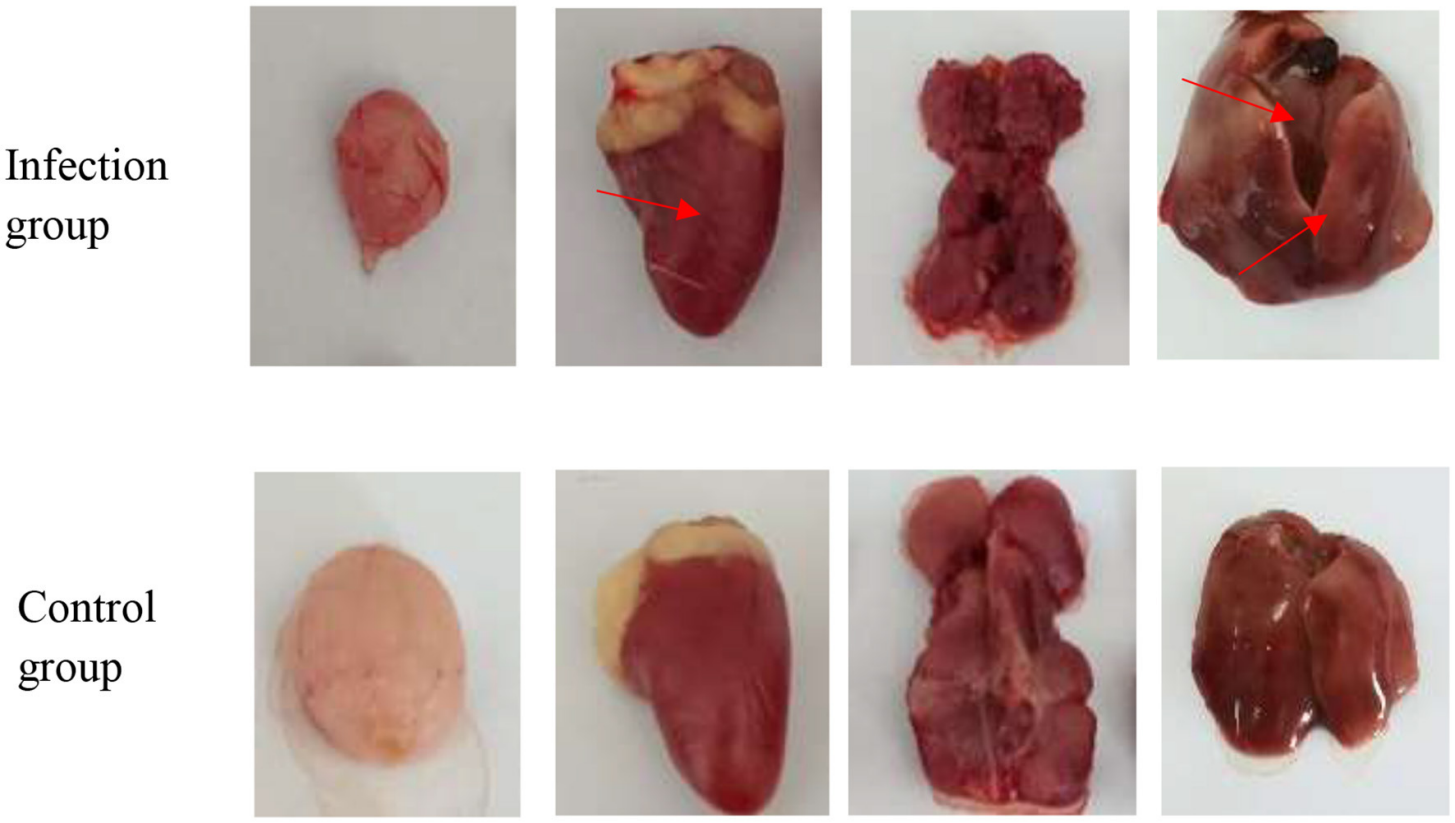
Gross observation of organs from 1-day-old SPF chickens infected with APV. Necropsy lesions observation of Bursa, Heart, Kidney, and Liver on the 1-day-old. SPF chickens infected with APV. The pathological features were observed in multiple organs.

**Figure 4C:**
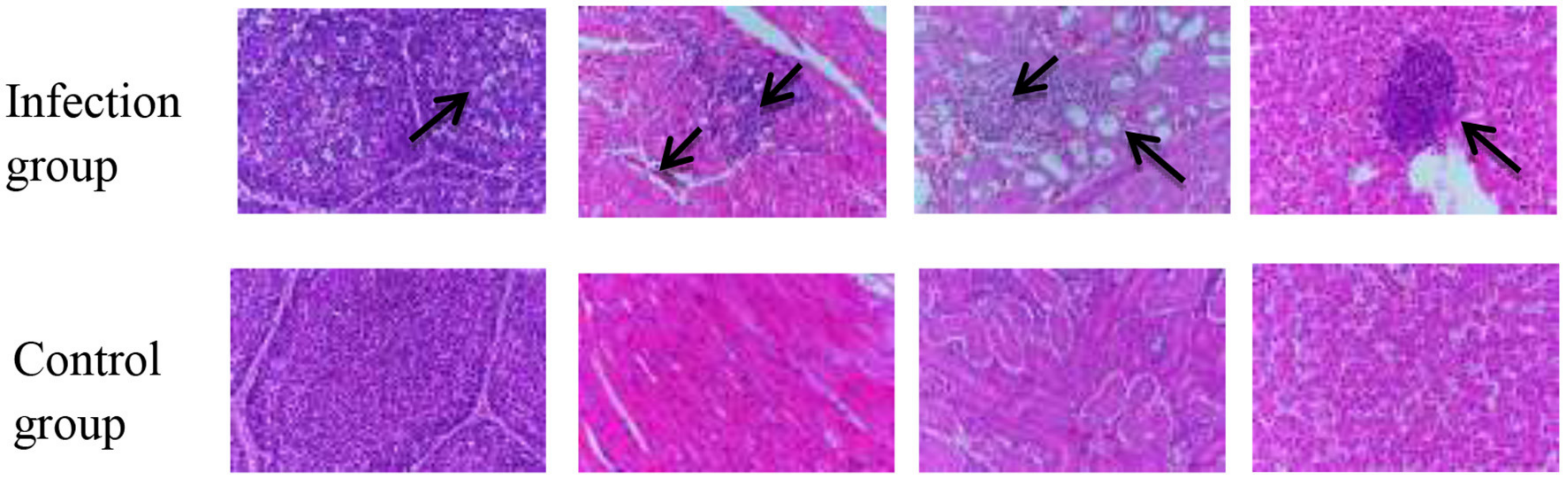
Histological sections of 1-day-old SPF chickens infected with APV showing typical lesions. Histopathological Observation of Bursa, Heart, Kidney, and Liver on the 1-day-old SPF chickens infected with APV. The pathological features were observed in the sections of multiple organs.

In the published article, there was an error in [Figure 4C] as published. [In Figure 4B, the macroscopic distribution of organs does not correspond to the histological images in Figure 4C (specifically liver and kidney).]. The corrected [Histological sections of 1-day-old SPF chickens infected with APV showing typical lesions.] and its caption ^**^[ Histopathological Observation of Bursa, Heart, Kidney, and Liver on the 1-day-old SPF chickens infected with APV. The pathological features were observed in the sections of multiple organs.] appear below.

We thank the reviewer for this important observation. Upon re-examination, we confirm that the histological images in Figure 4C are arranged in the order of bursa, heart, kidney, and liver, which is consistent with the order of organs shown in the macroscopic image of Figure 4B. The macroscopic image in Figure 4B and the histological images in Figure 4C are derived from representative cases of different 1-day-old SPF chickens infected with APV. However, we acknowledge that the presentation may have caused confusion. To clarify, we have [added a more detailed explanation in the figure legend, labeled the images more explicitly to indicate that they are from separate but representative cases]. We believe this revision improves the clarity of the data presentation.

In the published article, there was an error in [Table 5] as published. [The liver does not show the granulomas mentioned in Table 5]. The corrected [Basophilic intranuclear inclusions] and its caption ^**^[Granulomatous inflammation] appear below.

**Table 5:** Histopathological observation.

Group		Histopathological observation				Infections
		Bursa	Heart	Kidney	Liver	
Different days of age	1-day-old	germinal centers were lost, hyperplasia of connective tissue	Infiltration of macrophages and lymphocytes, interstitial myocardial hemorrhage	Interstitial nephritis, protein exudation from the lumen of the renal tubules, glomerulus atrophy	Intranuclear inclusions, granulomatous inflammation	**+**
	5-day-old	germinal centers were lost, hyperplasia of connective tissue	Infiltration of macrophages and lymphocytes	Interstitial nephritis, protein exudation from the lumen of the renal tubules	Intranuclear inclusions, granulomatous inflammation	**+**
	10-day-old	germinal centers were lost, hyperplasia of connective tissue	Infiltration of macrophages and lymphocytes	Interstitial nephritis	Granulomatous inflammation	**+**
	20-day-old	germinal centers were lost	Infiltration of macrophages and lymphocytes	Interstitial nephritis	Granulomatous inflammation	**+**
Different dose APV infection	10^6.0^TCID_50_	germinal centers were lost, hyperplasia of connective tissue	Infiltration of macrophages and lymphocytes, interstitial myocardial hemorrhage	Interstitial nephritis, protein exudation from the lumen of the renal tubules, glomerulus atrophy	Granulomatous inflammation, a large number of lymphocytic infiltrates	**+**
	10^5.0^TCID_50_	germinal centers were lost, hyperplasia of connective tissue	Infiltration of macrophages and lymphocytes	Interstitial nephritis	Swollen with marginal stasis	**+**
	10^4.0^TCID_50_	germinal centers were lost	Infiltration of macrophages and lymphocytes	Interstitial nephritis	Granulomatous inflammation	**+**
	10^3.0^TCID_50_	germinal centers were lost	/	Interstitial nephritis	/	**+**

This table counts and compares the histopathological observation of chickens infected with APV virus at different ages and with different doses of APV virus.

In the published article, there was an error. [Incorrect Terminology (Basic)].

A correction has been made to **[Discussion]**, [No.2]. This sentence previously stated:

“[Hair follicles may show hyperkeratosis]”

The corrected sentence appears below:

“[Feather follicles may show hyperkeratosis]”

In the published article, there was an error. [Incorrect Terminology (Basic)].

A correction has been made to **[Table 5]**, [Heart]. This sentence previously stated:

“[monocytes]”

The corrected sentence appears below:

“[macrophages]”

In the published article, there was an error. [Incorrect Terminology (Basic)].

A correction has been made to **[Result]**, [Pathogenicity in SPF chickens],[No.3]. This sentence previously stated:

“[H&E staining indicated that the germinal centers of bursal lymphatic nodules were lost]”

The corrected sentence appears below:

“[H&E staining indicated that the germinal centers of bursal lymphoid nodules were lost]”

In the published article, there was an error. [Incorrect Terminology (Basic)].

A correction has been made to **[Discussion]**, [No.3]. This sentence previously stated:

“[Some studies reported that balloon inflammation and septicemia were also observed]”

The corrected sentence appears below:

“[Some studies reported that ballooning change and septicemia were also observed]”

In the published article, there was an error. [Incorrect Terminology (Basic)].

A correction has been made to **[Result]**, [Pathogenicity in SPF chickens], [No.3]. This sentence previously stated:

“[Ocular lesion analysis of the tissues revealed the presence of normal lung tissue in the control group and significant lesions in the bursa of Fabricius, the kidney]”

The corrected sentence appears below:

“[Macroscopic observation of the lesions of the tissues revealed the presence of normal lung tissue in the control group and significant lesions in the bursa of Fabricius, the kidney]”

In the published article, there was an error. [Citation Misrepresentation].

A correction has been made to **[Discussion]**, [No.2]. This sentence previously stated:

“[Mononuclear cell infiltration is common in the vessel wall and around the vessels, while the endothelial cells of the vessels The lungs are less frequently affected and usually have a large number of hepatocytes]”

The corrected sentence appears below:

“[Mononuclear cell infiltration is common in the vessel wall and around the vessels, again containing inclusions, being present in the walls of some of the larger blood vessels]”

In the published article, there was an error. [Inaccurate Descriptions].

A correction has been made to **[Discussion]**, [No.2]. This sentence previously stated:

“[Histopathological observations reveal inclusion bodies in several organs, with basophilic granules present in the center of the inclusion bodies which show varying degrees of vascular degeneration]”

The corrected sentence appears below:

“[Histopathological observations reveal inclusion bodies in several organs, amphophilic to basophilic inclusion bodies with a clearing of the centre.]”

The original article has been updated.

